# Do gene expression changes in articular cartilage proteases of the synovial membrane correlate with expression changes of the same genes in systemic blood cells?

**DOI:** 10.1007/s00264-013-2195-8

**Published:** 2013-11-22

**Authors:** Adam Kwapisz, Michał Chojnacki, Marcin Domżalski, Andrzej Grzegorzewski, Marek Synder

**Affiliations:** 1 Department of Orthopaedics and Paediatric Orthopaedics, Medical University of Lodz, ul. Drewnowska 75, 91-002 Lodz, Poland; 2 Department of Medical Biochemistry, Medical University of Lodz, ul. Mazowiecka 6/8, 91-215 Lodz, Poland

**Keywords:** Osteoarthritis, Hyaline cartilage, Metaloproteinases, Agrecanases, ACL, Menisci

## Abstract

**Purpose:**

The aim of our study was to find whether an injury of the knee joint tissues increases gene expression of selected hyaline cartilage degenerating enzymes such as matrix metaloproteinases (MMP) and aggreacaneses (Agg).

**Methods:**

A total of 138 patients (81 female, 57 male) were admitted for knee arthroscopy with a mean age of 38.8 years. Full blood samples were collected preoperatively and synovium samples intraoperatively. Joint tissue lesions such as menisci, anterior cruciate ligament (ACL) and hyaline cartilage were estimated. Real time PCR with spectrophotometric analysis was performed.

**Results:**

An ACL lesion was found in 56 patients, medial menisci (MM) in 65, and lateral menisci (LM) in five. Chondral lesions were estimated according to Outerbridge’s grading system. In laboratory tests correlation between ACL tear and gene expression was seen except TIMP1 in serum (p < 0.05). In MM lesions MMP9, Agg2 elevation in serum was observed. LM lesions erased MMP13, MMP14 in serum and MMP8 in synovium. Chondral lesions revealed that many genes had higher expression in patients without hyaline degeneration. All of the gene expressions correlated between serum and synovium.

**Conclusion:**

An ACL lesion provokes elevation in expression of proteases genes, while the influence of other lesions remains elusive. Gene expression in synovium correlates with peripheral blood.

## Introduction

It is estimated that, during the year 1995, 40 million people were treated for osteoarthritis (OA) in the United States of America, with a simultaneous prognosis of the annual incidence of the disease to rise up to 59.4 patients in 2020 [1]. The risk factors for OA include, among others, practising sports and injuries of a given joint [2]. Gelber et al. demonstrated that, among subjects with a history of knee joint trauma, as many as 13.9 % of them presented with OA before the 65th year of life, while it was only 6 % in the control group [3]. Similarly, in the Framingham study, a five-fold higher risk of developing OA was estimated in subjects after knee joint trauma in history [4].

Osteoarthritis is associated with some prevalence of catabolic processes of the hyaline cartilage vs. its regenerative processes [5, 6]. These changes are controlled by inflammatory cytokines from the synovial membrane and chondrocytes, such as, for example, IL1 and TNF-α. They are present in synovial fluid and stimulate the secretion of other cytokines in addition to influencing the synthesis of articular cartilage damaging proteases [7–10]. Among the above-mentioned enzymes, the key role in articular cartilage destruction is played by adamlysins (including aggrecanases [Agg]) and matrix metalloproteinases (MMPs)[9, 10].

Tajima et al. [11] found that a post-traumatic haematoma could result from increased MMP2 and MMP9 levels, while Tchetvierikov observed increased proMMP1 levels in patients after joint trauma [12]. Some reports are also suggestive of MMP participation in the reconstruction process of the anterior cruciate ligament (ACL) [13]. It has been documented that MMP2 levels increase after ACL lesion [14]. Enhanced activity of MMP3 of aggrecanase 1 (Agg1) and 2 (Agg2) was also observed in degeneratively changed menisci [15, 16].

The goal of our study was to find out whether an injury of knee joint elements increases gene expression in selected proteases, cytokines and inhibiting factors (MMP1, MMP2, MMP8, MMP9, MMP13, MMP14, AGG1 and AGG2 proteases, TIMP1 and TIMP2—their inhibitors—and IL1 and TNFα cytokines). Moreover, we determined whether gene expression variations in the synovial membrane correlate with changes in peripheral blood cells.

## Material and methods

The study group consisted of 138 patients (81 female, 57 male) admitted for knee joint arthroscopy. In 29 of them, anterior cruciate ligament reconstruction was simultaneously performed. In 74 patients, the surgery was carried out in the right lower limb and in 64, it was the left knee joint. All the patients who qualified for the study were free of metabolic diseases, endocrine disorders, rheumatic and connective tissue diseases, hormonal contraception, steroid therapy, previous operations or fractures, with no history of nicotine, alcohol or drug addiction. The mean age in the study group was 38.8 years (median age, 35 years).

Following the qualification procedure into the study group, peripheral blood samples were collected from each patient on admission to the hospital. The blood was collected into 2.6-ml Monovette® EDTA KE test tubes. After 20 minutes at room temperature, the tubes with collected blood were frozen to a temperature of −20° C. The peripheral blood was collected from superficial vessels in the cubital fossa.

The anterolateral or anteromedial arthroscopic approach was selected in all patients. The knee joint was evaluated, using a four millimetre arthroscopic camera to assess the type and degree of possible lesions of the cruciate ligaments, menisci and the articular cartilage. Joint surface lesions were classified according to the Outerbridge scale [17]. Then, fragments of the ACL-surrounding synovial membrane were collected, either from approximately half of its length or directly from the region of its lesion. The collected material samples were immediately placed in sterile and capped 1.5-cm^3^ tubes with RNAlater® solution. Such processed samples were then cooled down to 4 °C and, on the second day, frozen to −20 °C.

The total cellular RNA was isolated from the synovial membrane and suspended in RNAlater® solution by the modified Chomczynski’s method using Trizol® (Invitrogen) reagent [18]. A similar procedure was applied to the peripheral blood samples from EDTA-containing tubes. The isolated RNA concentration was spectrophotometrically assayed, measuring absorbance at wave length of 260 nm. Purity of the isolates was evaluated by means of the A260/A280 coefficient, while RNA concentration and purity were measured with a Picodrop™ spectrophotometer. The obtained RNA solution was frozen at −80 °C and stored until further analysis.

The real-time reverse transcription-polymerase chain reaction (RT-PCR) method was applied to assess expression of the studied genes at the RNA level. The first stage in that method was a reverse transcription process in which complementary DNA (cDNA) was obtained and synthesised on mRNA array, with mRNA having been isolated from blood and tissues. The isolated RNA was then subjected to reverse transcription with an AccuScript High-Fidelity RT-PCR Kit (Agilent Technologies®). Real-time PCR is the second stage of the RT-PCR method.

A real-time PCR set, used in our study, encompassed FastStart Universal Probe Master (ROX) mixture, containing Taq DNA FastStart polymerase and a set of probes specific for amplified DNA fragment.

An RT-PCR analysis was carried out with a Stratagene Mx3005P instrument (Agilent Technologies). The relative mRNA expression was depicted with the 2-^ΔΔCt^ formula [19]. The GAPDH gene was used for internal control.

The expression of genes was evaluated in MMP1, MMP2, MMP8, MMP9, MMP13, MMP14, Agg1 and Agg2 proteases, TIMP1 and TIMP2—their inhibitors—and IL1 and TNFα cytokines.

A statistical analysis was performed by means of the Statistica software (Statsoft Inc., Licence No. SN AXAP911E504325AR-K). The level of statistical significance was established at p < 0.05. Distribution normality was verified by the Shapiro-Wilk test. Non-parametric tests (the U Mann-Whitney’s test and the Kruskal-Wallis one-way analysis of variance) were applied because of considerable deviations from normality. Differences among the subgroups were validated by the Mann–Whitney’s U test. Correlations were evaluated by the Spearman’s rank correlation tests.

The study protocol was approved by the Bioethical Commission (Approval No. RNN/125/09/KE).

## Clinical outcomes

**Fig. 1:**
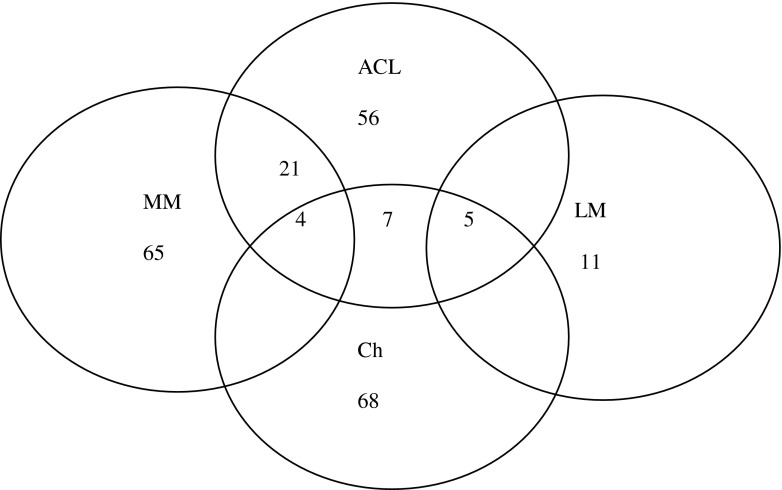
Distribution of intraoperatively identified knee joint lesions (ACL anterior cruciate ligament, LM lateral meniscus, MM medial meniscus, Ch articular cartilage)

An intraoperative evaluation of operated joints revealed ACL lesions in 56 patients. Medial meniscus (MM) lesions were diagnosed in 65 patients, whereas 11 patients demonstrated lateral meniscus (LM) damage. MM lesions were also observed in 21 and LM in five patients with an injured cruciate ligament. Joint surface evaluation revealed Grade I, according to Outerbridge’s grading system, in seven patients, grade II in 32, grade III in 25 and grade IV in four patients. Seventy patients did not show any hyaline cartilage abnormalities. Among the patients with affected hyaline cartilage, ACL tear was identified in 11 patients, out of whom, four also presented with MM injury. Meniscal lesions were found in 30 patients, being concomitant with cartilage surface injuries (Fig. 1).

## Laboratory tests

While analysing the effects of ACL injuries on the expression level of studied genes, the lack of any significant correlation was observed only in case of TIMP1 in peripheral blood. Regarding all the other genes, the level of their expression increases with ligament injury progressions (p < 0.05).

In patients with MM lesions, statistically significant changes were found for MMP9, Agg2 and TIMP2 in peripheral blood and MMP13 in the synovial membrane. We also observed that, while MMP9 and Agg2 expression levels were growing with MM injuries, the levels of MMP13 (synovial membrane) and TIMP2 (peripheral blood) were higher in patients with normal (unaffected) meniscus. LM injuries significantly increased MMP13 and MMP14 gene expression levels in peripheral blood. In addition, we noted higher expression levels of the MMP8 gene in synovial membranes of the patients with normal lateral meniscus vs. those with injured LM (p < 0.05).

Analysing the effects of articular surface injury, only a statistically significant increase of TIMP2 expression level was noted in both studied tissues, while no correlation was indicated, either for MMP13, MMP14 or TIMP1 in peripheral blood or for MMP8, MMP14 or TIMP1 in synovial membrane cells. In turn, the other studied genes demonstrated higher expression levels in the patients with normal hyaline cartilage.

In a subsequent stage of the study, we compared the effects of concomitant meniscus and articular cartilage injuries on gene expression levels in patients with torn ACL. Statistically significant injuries were observed in only nine enzymes. Regarding the synovial membrane, MMP1, MMP2 and MMP13 gene expression levels were lower in the patients with injuries of both anterior cruciate ligament and meniscus plus articular surface. Moreover, MMP2 demonstrated its lowest values also in the patients with torn ACL and injured articular cartilage. Additional injuries of intra-articular structures bring about significantly higher expression of the membranous MMP 8 and MMP9 genes. Isolated ACL injuries caused increased IL1 expression levels and significant decreases in TIMP2 expression levels in synovial membrane, as well as in MMP1 and MMP2 expression levels in peripheral blood cells. The other assays revealed statistically insignificant differences only (Table 1).

**Table 1:** Expression changes of the studied genes vs. the type of knee joint injury (p < 0.05; ↑ increase in affected subjects, ↓ increase in normal subjects, ↔ no statistical significance)

Test medium	ACL	MM	LM	Cartilage
Synovial membrane	All ↑	MMP13↑ the other ↔	MMP8↓ the other ↔	TIMP2↑ MMP8↔ MMP14↔ TIMP1↔ the other ↓
Peripheral blood	TIMP1 ↔ the other ↑	MMP9 ↑ Agg2 ↑ TIMP2↓ the other ↔	MMP13↑ MMP14 the other ↔	TIMP2↑ MMP13↔ MMP14↔ TIMP1↔ the other ↓

ACL anterior cruciate ligament, LM lateral meniscus, MM medial meniscus

In the final stage of the study, we checked whether the results, obtained for synovial membrane, corresponded with those for systemic blood cells. Significant correlations were confirmed among all the studied gene expressions. The strongest correlation was obtained for Agg1 (r = 0.89) and for MMP1 (r = 0.88) and MMP2 (r = 0.87). The weakest, but statistically significant, correlation was obtained for MMP8. The results indicate that, in the course of gene expression changes in synovial membrane, the same changes occurred in the peripheral blood for all the analysed genes (Table 2).

**Table 2:** Correlations between studied gene expression levels in the synovial membrane and blood (p < 0.05)

Comparison	Correlation, r	p
Synovium-MMP1 - Blood-MMP1	0.88	0.0000
Synovium-MMP2 - Blood-MMP2	0.87	0.0000
Synovium-MMP8 - Blood-MMP8	0.45	0.0000
Synovium-MMP9 - Blood-MMP9	0.60	0.0000
Synovium-MMP13 - Blood-MMP13	0.57	0.0000
Synovium-MMP14 - Blood-MMP14	0.73	0.0000
Synovium-Agg1 - Blood-Agg1	0.89	0.0000
Synovium-Agg2 - Blood-Agg2	0.84	0.0000
Synovium-IL1 - Blood-IL1	0.82	0.0000
Synovium-TNF - Blood-TNF	0.77	0.0000
Synovium-TIMP1 - Blood-TIMP1	0.58	0.0000
Synovium-TIMP2 - Blood-TIMP2	0,61	0.0000

## Discussion

It appears from the definition, established in Monterey, that osteoarthritis includes, among others, destabilisation of biochemical and molecular processes, which may lead to damage of the joint constituting tissues, resulting in pain disorders and gradually reduced mobility of the affected joint [20]. One of the aetiological factors, which may contribute to OA development, is the history of trauma. Gelbert et al. noted that 13.9 % of subjects with knee joint trauma in their history had presented with degenerative changes already before the 65th year of life, while it had been merely 6 % in the control group. [3] The Framingham study revealed a five-fold higher risk of osteoarthritis occurrence in patients after knee joint injury [4].

Monemdjou described MMP1 and MMP13 as the ‘‘major” mediators of articular cartilage degradation; additionally, Tchetvierikov observed increased proMMP1 levels in cases after articular trauma [10, 12]. In Tajim’s opinion, intra-articular haematoma may increase MMP2 and MMP9 levels [11]. This corresponds with our data, obtained in patients with injured ACL, where almost all gene expression levels were statistically increased. Zhou et al. also observed increased MMP2 levels in subjects with injured ACL [21]. Increased MMP1, IL6, TNFα and TIMP1 levels, observed in case of torn ACL, were also observed by Taskiran et al. [22] and by Higuchi et al. [23]. It should be noted that TIMP1 levels, assayed in the peripheral blood, did not show any statistically significant differences.

The assay of genes, performed by Foos for nine metalloproteinases of the extracellular matrix in ACLs, collected from cadavers, seems to be a fairly interesting observation. The author suggests that it may influence reconstruction of this ligament after trauma [13]. Our results may suggest very active processes of tissue metabolism in our patients with injured ACL, simultaneously validating Foos’ observations. The fact that only TIMP1 levels in peripheral blood were not increased in the examined patients with torn ACL may be regarded as additional evidence.

Taking into account the fact that ACL tear is often accompanied by injuries of other intra-articular tissues, the influence of concomitant injuries on expression levels of the studied genes was also evaluated. Following the conclusions from the report by Sun et al. [16], changes in meniscus structure result in changes within the entire knee joint. Ishihara reports increased activities of MMP3 and of Agg1 and Agg2 in meniscuses of subjects with OA symptoms [15]. Liao et al. [24] revealed upregulation of some protein expression in patients with meniscal lesions. Regarding the study group, similar, increased Agg2 levels were obtained only in the patients with injured MM. It should also be noted that the patients with normal (intact) meniscuses in our study demonstrated higher MMP13 expression levels vs. the patients with injured MM. It should be emphasised that MMP13 is regarded to be one of the metalloproteinases most important for OA development [10, 21, 25]. Also evaluated was the expression of genes of the inflammatory process cytokines. Regarding the patients with torn ACL, increased IL1 and TNFα expression levels were obtained in all the studied tissues. Similarly, increased levels of these enzymes in subjects after trauma have been described by Rutgers, who also emphasised the role of IL6, IL8 and IL10 [26]. We would like to draw attention to the fact that, in cases of injured meniscus and articular cartilage, no significant differences were observed, which corresponds to the report of Manicourt et al. [14], who found that TNFα levels do not correlate with cartilage degradation markers. In contrast to that view, Ji et al. [27] report that allele ‘A’ of TNFα may increase the risk of OA. Having analysed the effects of articular cartilage injury, we observed higher expression levels for the majority of protease genes in normal subjects, i.e., with intact articular cartilage vs. those with injured articular surfaces (Fig. 1).

In his study, Naito found that MMP3 and MMP9 levels did not correlate with the radiological stage of the disease. However, most of the authors report increased levels of proteolytic enzymes in subjects with osteoarthritis [10, 28, 29].

Similarly, El-Arman et al. [30] obtained positive correlations between enzyme levels in peripheral blood and in the knee joint. Also Poole et al., using 846 epitope, achieved a positive correlation between the levels of articular cartilage aggrecans in plasma and articular fluid [31]. Similarly, Fraser et al. described a correlation of local OA markers with systemic markers [32]. We would, however, like to emphasise the fact that the majority of literature reports describe enzyme level assays in the articular fluid, whereas we assayed expression levels of the genes corresponding to those proteins. According to these facts we suggest that further research with longer follow up should be conducted to establish whether genes expression elevations could be prognostic for OA in future.

## Conclusions

1. Gene expression levels in hyaline cartilage proteases of the synovial membrane correlate with peripheral blood cells.
2. Cruciate ligament injuries increase gene expression levels in hyaline cartilage proteases.
3. Possible effects of damage of other articular structures remain unclear, demanding further studies.
